# Development of a transformation system for *Hirsutella* spp. and visualization of the mode of nematode infection by GFP-labeled *H. minnesotensis*

**DOI:** 10.1038/srep10477

**Published:** 2015-07-20

**Authors:** Jingzu Sun, Sook-Young Park, Seogchan Kang, Xingzhong Liu, Junzhi Qiu, Meichun Xiang

**Affiliations:** 1 State Key Laboratory of Mycology, Institute of Microbiology, Chinese Academy of Sciences, No. 3 Park 1, West Beichen Road, Chaoyang District, Beijing 100101, China; 2 Department of Plant Pathology and Environmental Microbiology, Pennsylvania State University, University Park, PA 16802, USA; 3 Department of Life Science, Fujian Agriculture and Forestry University, No. 15 Shangxiadian Road, Cangshan District, Fuzhou 350002, China

## Abstract

*Hirsutella rhossiliensis* and *H. minnesotensis* are endoparasitic fungi of the second-stage juvenile (J2) of the soybean cyst nematode (*Heterodera glycines*) in nature. They also parasitize both *H. glycines* J2 and *Caenorhabditis elegans* on agar plates. *Agrobacterium tumefaciens*-mediated transformation conditions were established for these *Hirsutella* spp. The resulting transformants were similar to the corresponding wild-type strains. The infection processes of *H. glycines* J2 and *C. elegans* second larval stage (L2) by *H. minnesotensis* expressing ZsGreen were microscopically analyzed. Conidia of *H. minnesotensis* adhered to passing nematodes within 8 h post-inoculation (hpi), formed an infection peg between 8 and 12 hpi, and penetrated the nematode cuticle between 12 and 24 hpi for *C. elegans* L2 and between 12 and 32 hpi for *H. glycines* J2. Hyphal proliferation inside of the nematode coelom was observed at approximately 32 hpi for *C. elegans* L2 and at approximately 40 hpi for *H. glycines* J2. The fungus consumed the whole body and grew out to produce conidia at approximately 156 and 204 hpi for *C. elegans* L2 and *H. glycines* J2, respectively. The efficient transformation protocol and a better understanding of infection process provide a solid foundation for studying the molecular and cellular mechanisms underlying fungal parasitism of nematodes.

The soybean cyst nematode (SCN, *Heterodera glycines* Ichinohe) is one of the most destructive plant-parasitic nematodes of soybean in many regions of the world^1^,^2^. This nematode reduces yield both directly by siphoning off nutrients from infected plants and indirectly by creating wound sites that facilitate secondary fungal infection^3^. The endoparasitic fungi *Hirsutella rhossiliensis* and *H. minnesotensis* produce conidia that adhere to the cuticles of passing nematodes. Attachment leads to hyphal penetration and eventually to nematode death^4^,^5^. Only conidia that are attached to conidiogenous cells are infectious^6^. Once successful penetration occurs, they proliferate in the nematode body, filling it with mycelia, and produce new conidia within a few days to initiate a new infection cycle. Three endoparasitic *Hirsutella* spp. have been identified to date, including *H. rhossiliensis* Minter & Brady^4^, *H. minnesotensis* Chen, Liu & Chen^7^, and *H. vermicola* Xiang & Liu^8^. *H. rhossiliensis* has been detected worldwide^1^,^9^,^10^ and infects a wide range of hosts, including nematodes in the genera *Heterodera*, *Meloidogyne*, *Xiphinema*, and *Rotylenchus* and soil mites^11^,^12^. *H. minnesotensis* has been found in the U.S., Germany, Poland and China^12^,^13^, and it also infects diverse nematodes in the genera *Aphelenchoides*, *Heterodera*, *Mesocriconema*, *Belonolaimus*, *Hoplolaimus*, *Steinernema*, and *Heterorhabditis*^12^,^14^ and Collembola^15^. *H. vermicola* was recently discovered in bacteria-feeding nematodes^8^.

Because second-stage juveniles (J2s) of *H. glycines* are infectious, their presence in soil is directly associated with soybean yield reduction^16^. Both *H. rhossiliensis* and *H. minnesotensis* parasitize high percentages of SCN juveniles in diverse agricultural soils, supporting their potential as biocontrol agents. The *H. rhossiliensis* isolate OWVT-1 can reduce the density of *H. glycines* eggs by 95% and the J2 density by 98% in pots containing field soil^17^. Both species have also displayed high efficiencies in suppressing *H. glycines* density in greenhouse experiments^18^. As the dominant parasite of *H. glycines* J2 in China, *H. minnesotensis* is considered a main contributor to the suppression of SCN in field soils^19^. Field trials have demonstrated its efficiency for SCN biological control^20^.

Enhanced understanding of the infection process of *H. glycines* by *H. minnesotensis* is crucial to exploit this fungus as an effective biocontrol agent and also to study the molecular mechanisms underlying its interaction with nematodes. *Caenorhabditis elegans* has been widely used as a model organism to study diverse fundamental biological processes, mainly because of its experimental tractability (e.g., easy manipulation on agar, short generation time, and well-established experimental tools) and rich resources, such as genome sequences and diverse mutants^21^. Although the parasitism of bacteria-feeding nematodes, such as *C. elegans* by *H. minnesotensis* has not been detected in nature, the successful infection of *C. elegans*, by this fungus at different larval stages on agar plates has been documented^22^, suggesting that this model nematode can potentially help enhance the current understanding of the molecular and cellular mechanisms underlying *H. minnesotensis*–nematode interactions. To realize this potential, it is critical to develop genetic manipulation tools for *H. minnesotensis*.

Fluorescent proteins have been expressed in a wide variety of organisms, including bacteria, fungi, plants, and animals^23^, and have facilitated the imaging of diverse organismal interactions and cellular processes^24^,^25^,^26^. In this study, we established an *Agrobacterium tumefaciens*-mediated transformation (ATMT) protocol for *H. rhossiliensis* and *H. minnesotensis* and generated transformants expressing fluorescent proteins with non-overlapping spectral properties. One *H. minnesotensis* transformant was used to investigate the manner by which it infects *H. glycines* J2s and *C. elegans* L2s using fluorescence microscopy.

## Results

### Transformation of *Hirsutella* spp. with three fluorescent protein genes

**Figure 1:**
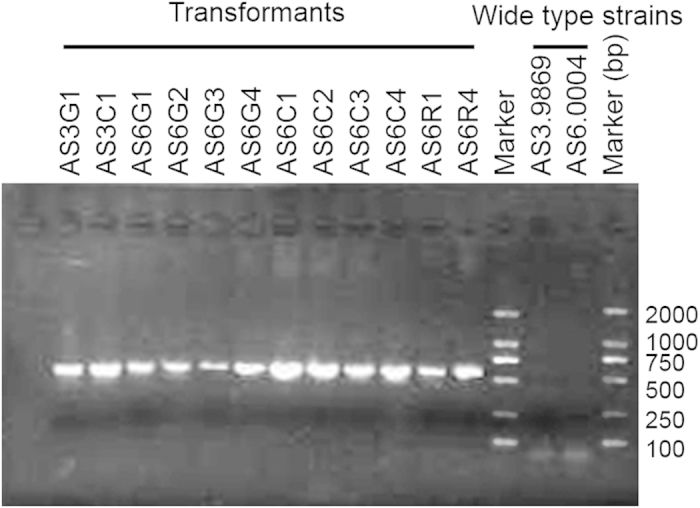
Detection of the *hph* gene in twelve randomly selected transformants using PCR. The presence of the *hph* gene in transformants of *H. minnesotensis* AS3.9869 and *H. rhossiliensis* AS6.0004 was detected using PCR. This PCR product was absent in AS3.9869 and AS6.0004.

**Figure 2:**
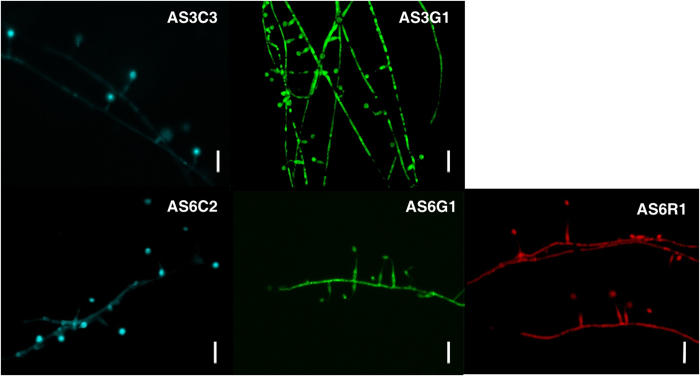
Transformants of *H*.*minnesotensis* AS3.9869 and *H*.*rhossiliensis* AS6.0004 expressing a fluorescent protein. Expression of cyan (AsCyan) and green (ZsGreen) fluorescent proteins was detected in hyphae and conidia of transformants AS3C3 and AS3G1 of *H*. *minnesotensis* AS3.9869, respectively. Expression of AsCyan, ZsGreen and AsRed, a red fluorescent protein, in hyphae and conidia of transformants AS6C2, AS6G1 and AS6R1 of *H. rhossiliensis* AS6.0004, respectively, by slide culture are shown. Scale bar = 20 μm.

The sensitivities of *Hirsutella* spp. to hygromycin B and geneticin were determined. The minimum concentrations of hygromycin B and geneticin resulting in complete growth inhibition were 150 μg/ml and 400 μg/ml, respectively, for *H. minnesotensis* AS3.9869 and 100 μg/ml and 400 μg/ml, respectively, for *H. rhossiliensis* AS6.0004. Growth responses of these isolates to hygromycin B on potato dextrose agar (PDA) and cornmeal agar (CMA) were indistinguishable. Based on their higher sensitivity to hygromycin B than geneticin, we chose the *hph* gene, which confers resistance to hygromycin B, to select transformants. Binary vectors containing *hph* and the *AsRed, AsCyan* or *ZsGreen* gene were used for transformation. PCR analysis of 12 randomly selected transformants revealed that the *hph* gene in the inserted T-DNA was detected in all selected transformants but not in the wild-type (wt) AS3.9869 or AS6.0004 strains (Fig. 1). Microscopic observations of slide cultures showed that all three fluorescent proteins were expressed strongly in hyphae and conidia of AS3.9869 and AS6.0004 transformants (Fig. 2).

**Figure 3:**
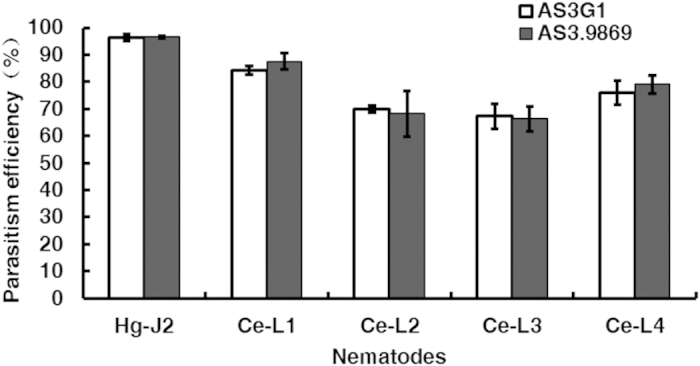
Efficiencies of parasitizing nematodes by *H*.*minnesotensis* AS3.9869 and its transformant AS3G1. The percentages of nematodes parasitized by AS3.9869 and AS3G1 were analyzed 24 hours after inoculation, which showed no significant differences in their ability to parasitize *H. glycines* J2 (Hg-J2) and four different larval stages of *C*. *elegans* (Ce-L1, Ce-L2, Ce-L3, and Ce-L4). Error bar indicates the standard deviations within three replications.

Morphology, growth characteristics and the capability to parasitize nematodes were evaluated for 50 transformants (10 *ZsGreen* and 10 *AsCyan* transformants of AS3.9869; and 10 *ZsGreen*, 10 *AsCyan* and 10 *AsRed* transformants of AS6.0004). Compared with the corresponding wt strains, several transformants parasitized fewer nematodes, which may be due to genetic changes that occurred during transformation. However, most transformants showed no significant differences from the wt strains. The transformant AS3G1, a *ZsGreen* transformant of *H. minnesotensis* AS3.9869, was selected to examine the manner by which it infects *C. elegans* and *H. glycines* (Figs. 2 and 3). Both AS3.9869 and AS3G1 parasitized substantial percentages of *H. glycines* J2 and *C*. *elegans* at four larval stages (L1-L4). The percentages of colonization for *H. glycines* J2 and *C. elegans* L1-L4s were 96.5%, 87.6%, 68.3%, 66.4% and 79.1%, respectively, for AS3.9869 and 96.4%, 84.3%, 69.9%, 67.2% and 78.0%, respectively, for AS3G1. There were no significant differences detected between AS3.9869 and AS3G1 (t_*0.05/2*_ = −0.715; *p* = 0.485). However, as shown in Fig. 3, the percentage of parasitized nematodes differed significantly between *H. glycines* J2 and *C. elegans* L1-L4 (*F* = 62.56; *df* = 4, 25; *p* < 0.001).

### Infection cycle of nematodes by *H. minnesotensis*

The infection cycle consists of four stages: recognition and adhesion; penetration; consumption of the nematode body; and sporulation^27^. Notable features observed at each of these stages of nematode colonization by AS3G1 are outlined below.

### Recognition and adhesion

**Figure 4:**
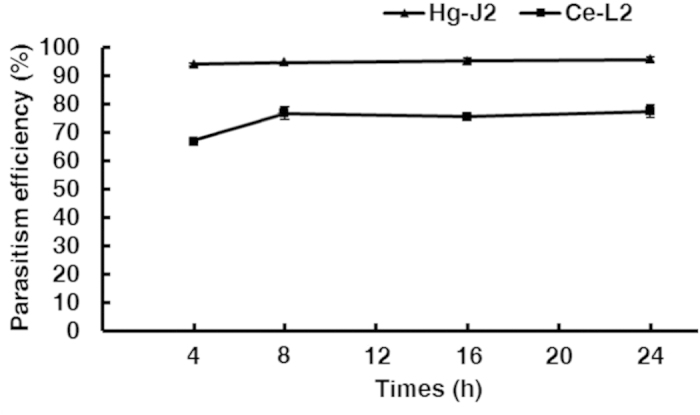
Percentages of nematodes infected by *H*.*minnesotensis* AS3.9869 and its transformant AS3G1 within 24 h post inoculation. Parasitism efficiencies of Hg-J2 and Ce-L2 by these strains at 4, 8, 16, and 24 h post inoculation are shown. Error bar indicates the standard deviation within three replications.

**Figure 5:**
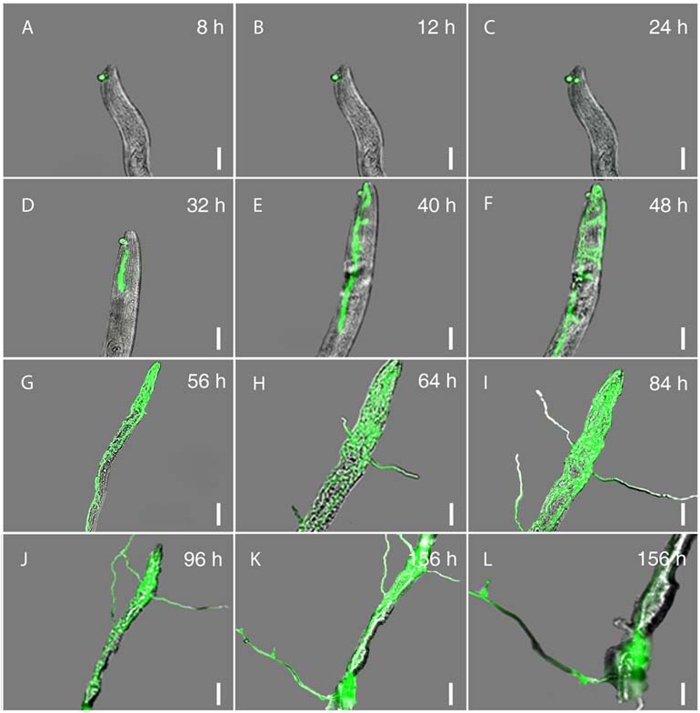
Progression of *H. glycines*J2 infection by *H*.*minnesotensis*. A, a spore of AS3G1 (a *H*. *minnesotensis* transformant expressing ZsGreen) attached to *H. glycines* second stage juvenile (J2); B, formation of an infection peg; C, the apex of the infection peg becoming globose; D, initial colonization of coelom by hyphae; E-G, hyphal growth through coelom; H-J, hyphal growth out of coelom; K-L, formation of new conidia. The scale bar in A-F, H-I, and L corresponds to 20 μm, whereas the scale bar in G, J and K equals 40 μm.

**Figure 6:**
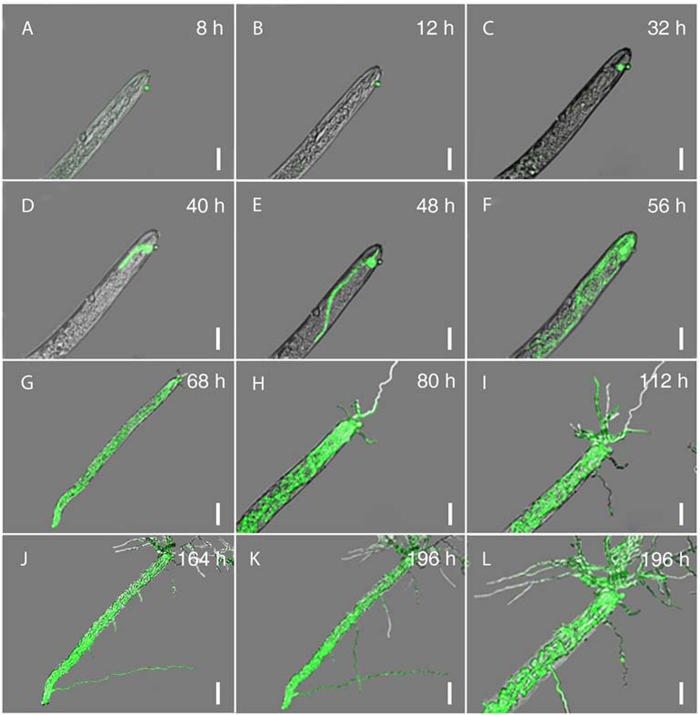
Progression of *C*.*elegans* L2 infection by *H*. *minnesotensis*. A, a spore of AS3G1 attached to *C*. *elegans* second stage larvae (L2); B, formation of an infection peg; C, the apex of the infection peg becoming globose; D, initial colonization of coelom by hyphae; E-G, hyphal growth through coelom; H-J, hyphal growth out of coelom; K-L, formation of new conidia. The scale bar in A-F, H-I, and L corresponds to 20 μm, whereas the scale bar in G, J and K equals 40 μm.

**Figure 7:**
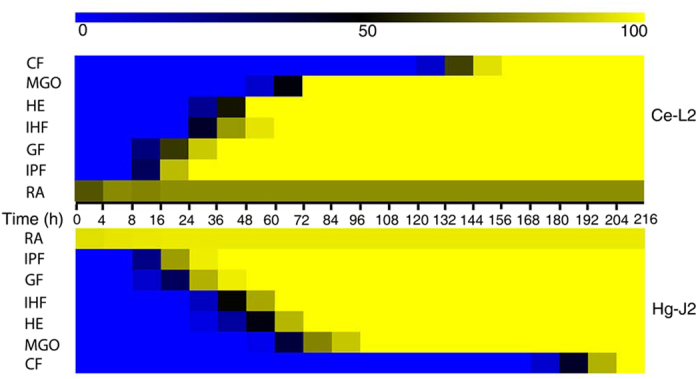
Comparison of the infection cycle of *H. glycines* J2s and*C*.*elegans* L2s by *H*.*minnesotensis* AS3G1. A heatmap was used to facilitate the comparison of the infection cycle of *H. glycines* J2s (Hg-J2) and *C*. *elegans* L2s (Ce-L2) by *H. minnesotensis* on agar plates. Patterns observed in 30 individuals for each nematode species were used to draw the map. The background color, changing from blue to black to yellow, indicates the percentage of nematodes parasitized at individual infection stages at the given observation point. The infection stages include recognition and adhesion (RA), infection peg formation (IPF), globus hyphal formation (GHF), initial hyphal formation (IHF), hyphal extension (HE), mycelia growing out (MGO) and conidial formation (CF). The infection cycle of Ce-L2s and Hg-J2s analyzed here were 156 h and 204 h, respectively.

Interactions of AS3G1 with *H. glycines* J2s and *C. elegans* L2s at 4, 8, 16, and 24 h post inoculation (hpi) showed that even at 4 hpi, more than 90% of *H. glycines* J2s had attached conidia (Fig. 4). The percentage of *C. elegans* L2s with attached conidia at this stage was approximately 65%, which was significantly lower than that observed for the *H. glycines* J2s. The percentage of parasitized *H. glycines* J2s did not vary greatly during this period (Fig. 4), suggesting that unattached *H. minnesotensis* conidia quickly recognized and adhered to passing *H. glycines* J2s. There was a noticeable increase in the colonization of *C. elegans* L2s between 4 and 8 hpi, after which it remained stable at approximately 75%. More conidia adhered to individual nematodes as time progressed, with up to 20 adhered spores for each nematode until mobility was lost. These results suggest that the stage of recognition and adhesion was mostly completed within 8 hpi for both nematodes (Fig. 5, 6, 7).

### Penetration

Following the germination of the conidia adhered to the nematodes, an infection peg was formed, and penetration of the nematode cuticle occurred between 8 and 12 hpi (Figs. 5, 6, 7). After penetration, the apex of the infection hypha became globus between 12 and 24 hpi for *C. elegans* L2 (Fig. 5C, Fig. 7) and between 12 and 32 hpi for *H. glycines* J2 (Fig. 6C, Fig. 7). Although *C. elegans* L2s were alive and could move slowly at 24 hpi, more than 80% of them were observed to be penetrated by one or more conidia. *H. glycines* J2s appeared dead at 32 hpi because their stylets were no longer moving, and more than 90% of them were penetrated by one or more conidia during this period (Fig. 7).

### Consumption of the nematode body

Once the fungus penetrated the nematode, the initial hypha started to grow in the nematode body. The fungus began to develop an initial hypha inside of *C. elegans* L2 (Fig. 5D) at 32 hpi and inside of *H. glycines* J2 at 40 hpi (Fig. 6D). Eighty percent of the examined nematodes with developed initial hyphae were found at 48 hpi for *C. elegans* L2 and at 60 hpi for *H. glycines* J2. The initial hyphae quickly extended inside of *C. elegans* L2 (Fig. 5E-G) and *H. glycines* J2 (Fig. 6E-G), and the coeloms of both nematodes were completely filled with mycelia at 84 hpi (Fig. 7).

### Sporulation

Hyphae began growing out of the colonized nematode coelom at approximately 56 hpi for *C. elegans* L2 (Fig. 5G) and at 68 hpi for *H. glycines* J2 (Fig. 6G), and hyphae were clearly visible at 64 hpi for *C. elegans* L2 (Fig. 5H-J, Fig. 7) and at 80 hpi for *H. glycines* J2 (Fig. 6H-J, Fig. 7). Subsequently, the fungus produced new conidia (Fig. 5K,L, Fig. 6K,L) from up to 94% of *C. elegans* L2s at 156 hpi and 84% of *H. glycines* J2s at 204 hpi (Fig. 7). In summary, the infection cycles of *C. elegans* L2 and *H. glycines* J2 by *H. minnesotensis* on agarose slides were completed in approximately 156 and 204 h, respectively.

## Discussion

Green fluorescent protein (GFP) and its color variants as molecular labels have been applied to studying diverse filamentous fungi^28^. To assess the interactions between nematophagous fungi and nematodes, attempts have been made to transform *Pochonia chlamydosporia*^23^, *Clonostachys rosea*^26^ and *Dactylellina cionopaga*^29^ with a gene encoding GFP. However, no *gfp* transformant of *P. chlamydosporia* was obtained^23^ because of its slower growth and limited sporulation, and the material coating its spores resulted in a very low transformation efficiency^26^. The goal of our study was to establish a robust transformation protocol for the dominant nematode endoparasitic fungi *Hirsutella* spp. in nature using fluorescent protein genes as markers to assess the manner by which they infect nematodes. ATMT was successful in transforming both *H. rhossiliensis* and *H. minnesotensis*, and most transformants expressed three fluorescent proteins with high efficiency and displayed growth, morphology, and pathogenicity that were indistinguishable from the corresponding wt strains. These transformants will facilitate ecological studies, such as the monitoring of the spatial and temporal distribution of *Hirsutella* spp. in soil and plant roots, competition between *H. rhossiliensis* and *H. minnesotensis*, and the elucidation of the molecular and cellular mechanisms of fungal interactions with nematodes.

Due to the movement of free-living nematodes, their microscopic observation is challenging. Conventional techniques for immobilizing nematodes include the uses of cyanoacrylate glue^30^ and anesthetic compounds, such as sodium azide (a metabolic inhibitor)^31^ and levamisole (a cholinergic agonist)^32^. Recently, a computer-controlled micro-fluidic device was developed to limit nematode mobility^33^. Although these methods have been successfully applied, some limitations remain. The use of the glue is labor-intensive and low-throughput. Anesthetic compounds have negative effects on *H. minnesotensis* growth. Further, microfluidic device cannot provide enough air for this fungus. Here, we successfully used the agarose fixation method to immobilize *H. glycines* J2 and *C. elegans* larvae without negatively affecting fungal infection.

*H. minnesotensis* is a cold-adapted fungus that preferentially parasitizes cyst nematodes, even in the presence of bacterial-feeding nematodes in nature^19^,^34^. Both *H. minnesotensis* and *H. rhossiliensis* can parasitize various nematodes, including *C. elegans,* on agar plates^15^,^22^. However, they cannot parasitize bacterial-feeding nematodes in natural soil, or *C. elegans* in autoclaved soil, as demonstrated in our recent study. The differences in parasitism between agar plates and soil suggest that some soil factors may interfere with fungal recognition and/or colonization of nematodes. Follow-up studies to address this supposition will be conducted using the transformants generated in this study.

Several characteristics of *C. elegans*, including its small size, short generation time, invariant developmental lineage, and sequenced genome, along with the powerful genetic manipulation tools available for its analysis and its ease of handling, have made it a model organism for studying the mechanisms underlying bacterial and fungal pathogenesis in animals and innate immunity^35^. Studies with bacterial pathogens, such as *Pseudomonas, Salmonella,* or *Serratia,* have been highly fruitful in revealing the existence of an innate immune system in *C. elegans,* similar to that in vertebrates^36^. This model organism has also been used to study innate immune responses to fungal infections^37^,^38^. Interactions between nematophagous fungi and nematodes have not been extensively studied.

The capability of *H. minnesotensis*, a dominant *H. glycines* J2 parasite, to infect *C. elegans* on agar plates presents a new model system for investigating the molecular and cellular mechanisms underlying the pathogenesis of nematophagous fungi and host defenses at different infection stages. The infection process of *H. minnesotensis* against *H. glycines* J2 and *C. elegans* L2 observed in this study has guided transcriptomics analyses of *C. elegans* L2 parasitized by *H. minnesotensis*^39^, as well as an ongoing study of the experimental evolution of parasitism of *Hirsutella* spp.

## Materials and Methods

### Strains and nematodes

*H. minnesotensis* AS3.9869 (strain HLJ1-10) was isolated from *H. glycines* J2 collected in Jiamusi, Heilongjiang, China in 2006. *H. rhossiliensis* AS6.0004 (strain OWVT-1) was isolated from *H. glycines* J2 collected in Waseca, Minnesota, USA in 1997^1^. Single-spore isolates of these fungi that parasitized a high percentage of *H. glycines* J2s^18^ were used for transformation. These strains were cultured on PDA at 25 °C and maintained in 15% glycerol at −80 °C until needed.

Three *A. tumefaciens* strains, including SK2245 (*A. tumefaciens* strain EHA105 that carries a pBHt2 binary vector containing the *AsRed* gene), SK2247 (EHA105 that carries a pBHt2 binary vector containing the *AsCyan* gene) and SK2251 (EHA105 that carries a pBHt2 binary vector containing the *ZsGreen* gene), were used for fungal transformation. The expression of all three fluorescent protein genes was driven by the promoter of the *Fusarium verticillioides* elongation factor 1α gene. These *A. tumefaciens* strains were cultured at 28 °C for 48 h in minimal medium^40^ supplemented with 50 μg/ml kanamycin before transformation.

Soybean cyst nematode race 4 was isolated from a soybean field near Beijing. For *H. glycines* egg preparation, newly formed cysts in soybean roots cultivated in the greenhouse were extracted using the sucrose floatation and centrifugation method^18^. Individuals at the J2 stage were prepared as described by Liu and Chen^1^ and used immediately.

Bristol strain N2 of *C. elegans* in mixed-stages was cultured on nematode growth medium (NGM) plates and seeded with *Escherichia coli* stain OP50^41^ at 24 °C. Fresh *C. elegans* specimens at the four larval stages were harvested following the method described by Xie *et al.*^42^ and used immediately.

### Preparation of fungal spores

Spores of *H. minnesotensis* AS3.9869 and *H. rhossiliensis* AS6.0004 were harvested by the scraping of 15-day-old cultures on PDA after the flooding of plates (5 plates per strain) with 15 ml of sterile water. After passing individual spore suspensions through two layers of Miracloth (Calbiochem, Cat. #475855, La Jolla, CA) to remove large debris, they were centrifuged at 10,000 rpm for 10 min and washed twice with sterile distilled water. The harvested spores were re-suspended in sterile water to produce a solution of 1 × 10^6^ spores/ml.

### Fungal transformation

To determine the sensitivities of the fungal strains to antibiotics prior to transformation, PDA and CMA plates with different amounts of hygromycin (50, 100, 150, 200, 250 μg/ml) or geneticin (50, 100, 200, 400, 800 μg/ml) and control plates without antibiotics were prepared. Small amounts of mycelia from the tested strains were inoculated on each plate and incubated for 15 days. The amounts of antibiotics that completely inhibited fungal growth were chosen for transformant selection. *Agrobacterium tumefaciens*-mediated transformation was performed according to Khang *et al.*^40^ with minor modifications. Each of the three *A. tumefaciens* strains described above was incubated in 1 ml of minimal medium (MM) supplemented with kanamycin (50 μg/ml) for 1-2 days at 28 °C. The resulting *A. tumefaciens* cultures were inoculated into induction medium (IM) at OD_600_ = 0.15. After the culturing of *A. tumefaciens* cells for 6 h at 28 °C at 200 rpm, a 100-μl suspension of *A. tumefaciens* cells and 100 μl of fungal spores (1 × 10^6^ spores/ml) were mixed and spread onto a nitrocellulose membrane (Whatman Cat. #7141 104; 47-mm diameter; 0.45-μm pore size) placed on co-cultivation medium in the presence of 200 μM AS. After 2 days of incubation at room temperature in the dark, the membrane was transferred to CMA selection medium containing hygromycin B (150 μg/ml for AS3.9869 and 100 μg/ml for AS6.0004) and cefotaxime (0.2 mM). The membrane was incubated for 2-3 weeks at room temperature until colonies formed.

Putative hygromycin B-resistant colonies were transferred to 24-well plates containing CMA selection medium with hygromycin B using sterile toothpicks. After the plates were incubated at 25 °C for 10 days, individual wells were flooded with sterile water. The water was pipetted up and down several times to dislodge the conidia. The conidia were spread onto water agar plates with hygromycin B and incubated for 24 h, and 10 single germinating spores for each transformation were then picked up with a sterile needle under a microscope and transferred to PDA plates for subsequent analysis and preservation.

### Characterization of putative transformants

A simple slide culture technique was used for detecting the fluorescence of the growing transformants. A PDA agar block (approximately 4 × 4 mm^2^ and 4 mm thick) inoculated with fungus was placed on a sterile slide with a coverslip^43^. The slide culture was placed into a 90-mm Petri dish sealed with parafilm to avoid contamination and to maintain humidity. After 7-10 days of incubation at room temperature, the mycelia were observed with a Zeiss Axio Imager A1 fluorescent microscope (Carl Zeiss, Germany) with AxioVision 4.6 software to confirm the expression of each of the three fluorescent proteins. Three filter sets were used as follows: (i) excitation at 460-480 nm and emission at 505-530 nm for ZsGreen; (ii) excitation at 540-552 nm and emission at 575-640 nm for AsRed; and (iii) excitation at 365 nm and emission at 420-470 nm for AsCyan.

Twelve randomly selected fluorescent transformants were cultured on cellophane membranes placed on PDA plates containing hygromycin B at 25 °C for 10 days. Mycelia were harvested from the cellophane, frozen in liquid nitrogen and ground to a fine powder. Fungal DNA was extracted using the CTAB method and purified using a Wizard® Genomic DNA Purification Kit (Promega, USA) according to the manufacturer’s instruction. The primers hph-F (5’-GAGCCTGACCTATTGCATCTC) and hph-R (5’-CCGTCAACCAAGCTCTGATAG) were used for PCR analysis to confirm the presence of the integrated *hph* gene following the protocol recommended by White and Chen^44^, with an initial denaturation step at 95 °C for 5 min, 35 cycles of denaturation at 95 °C for 30 s, annealing at 55 °C for 30 s, and elongation at 72 °C for 30 s and a final elongation step at 72 °C for 10 min. PCR products were separated in a 1% agarose gel in TAE buffer. The D2000 DNA ladder (TIANGEN Biotech, China) was used as a size marker.

### Evaluation of transformant stability and capacity to parasitize nematodes

Single-spore purified transformants were cultured on PDA plates without hygromycin B for at least five generations, and they were then evaluated for the stable production of individual fluorescent proteins.

To assess the pathogenicity of the selected transformants, wt strains and their transformants were inoculated on PDA plates at 25 °C. Individual conidial suspensions were prepared by washing 2-week-old colonies with sterilized 0.1% Tween-20 solution. The number of conidia was determined using a hemacytometer, and the conidial concentration was adjusted to 2×10^5^ spores/ml. An aliquot of the conidial suspension (200 μl) was spread onto the surface of each CMA plate. The plates were left uncovered for 30 min in a laminar flow hood to allow excess water to evaporate. After 2 weeks of incubation at room temperature, approximately 300 nematodes in 100 μl of 4.5 mM KCl solution were added to the center of each plate^45^. After 3 days of incubation, the nematodes were washed off using 5 ml of 0.1% Tween-20 solution. The percentage of nematodes with attached conidia or of those colonized by each strain was determined by observing 100 randomly chosen nematodes from each plate using an Olympus CK 40 microscope (Olympus, Japan) at 40–200× magnifications^18^. Four plates were used for each strain.

### Microscopic observation of infection process

To determine the effect of inoculation time on the percentage of parasitism by *H. minnesotensis*, 200 *H. glycines* J2s or *C. elegans* L2s were inoculated in each well of a 12-well plate containing 2-week-old fungal colonies growing on CMA at 25 °C. The nematodes were washed out with 1 ml of sterile 0.1% Tween-20 at different time points (4, 8, 16, and 24 h). The nematodes that were parasitized or possessed adhered conidia were counted.

After 4 h of incubation, the nematodes from each well of the 12-well tissue culture plates were collected using sterile water and transferred into each well of a 6-well tissue culture plate to observe parasitism and to select nematodes with a single conidium attached. One drop of 4% boiled agarose was placed on the center of a sterilized slide, and a sterilized coverslip was placed onto the agarose drop immediately and pressed down to spread the agarose^46^. Once the agarose was solidified, the coverslip was removed. One nematode with a single spore attached was picked up and transferred to the center of the agarose pad and covered with a coverslip. Five slides were prepared for each nematode species. The location of the nematode was marked, and three edges of the coverslip were sealed with Vaseline. Another 30 nematodes with single or multiple spores attached were picked up in the same manner and transferred to 5 different agarose pads as described above. The slides were kept in a container at a relative humidity of 98% (saturated solution of K_2_SO4)^47^ at 20 °C. The infection process was observed and photographed with a Zeiss fluorescence microscope (Axio Imager A1) with Apochromat 20× and 40× objective lenses, and AxioVision 4.6 software was used for image acquisition.

### Statistical analysis

The effects of the nematode species, stages and fungal species and isolates on the percentages of parasitized nematodes were determined using analysis of variance (ANOVA). The significances of the differences between the percentages of nematodes parasitized by AS3.9869 and AS3G1 were analyzed using the *t*-test. The SPSS (Statistical Package for the Social Sciences) software package (SPSS 16.0) was used for all statistical analyses. The data pertaining to the infection cycles of *H. minnesotensis* in *C. elegans* L2 and *H. glycines* J2 were analyzed using the MeV software package (MeV 4.8.1).

## Additional Information

**How to cite this article**: Sun, J. *et al.* Development of a transformation system for *Hirsutella* spp. and visualization of the mode of nematode infection by GFP-labeled *H. minnesotensis.* *Sci. Rep.* **5**, 10477; doi: 10.1038/srep10477 (2015).
