# Corrigendum: Development of a transformation system for *Hirsutella* spp. and visualization of the mode of nematode infection by GFP-labeled *H. minnesotensis*

**DOI:** 10.1038/srep14889

**Published:** 2015-10-08

**Authors:** Jingzu Sun, Sook-Young Park, Seogchan Kang, Xingzhong Liu, Junzhi Qiu, Meichun Xiang

Scientific Reports 5: Article number: 10477; 10.1038/srep10477 published online: 07202015; updated: 10082015.

This Article contains typographical errors in the legends of Figs 5 and 6.

In Fig. 5:

**Progression of** ***H. glycines*** **J2 infection by** ***H***.***minnesotensis***.

A, a spore of AS3G1 (a *H*. *minnesotensis* transformant expressing ZsGreen) attached to *H. glycines* second stage juvenile (J2); B, formation of an infection peg; C, the apex of the infection peg becoming globose; D, initial colonization of coelom by hyphae; E–G, hyphal growth through coelom; H–J, hyphal growth out of coelom; K–L, formation of new conidia. The scale bar in A–F, H–I, and L corresponds to 20 μm, whereas the scale bar in G, J and K equals 40 μm.

should read:

**Progression of** ***C. elegans*** **L2 infection by** ***H***.***minnesotensis***.

A, a spore of AS3G1 (a *H. minnesotensis* transformant expressing ZsGreen) attached to *C. elegans* second stage larvae (L2); B, formation of an infection peg; C, the apex of the infection peg becoming globose; D, initial colonization of coelom by hyphae; E–G, hyphal growth through coelom; H–J, hyphal growth out of coelom; K–L, formation of new conidia. The scale bar in A–F, H–I, and L corresponds to 20 μm, whereas the scale bar in G, J and K equals 40 μm.

In Fig. 6:

**Progression of** ***C***.***elegans*** **L2 infection by** ***H***. ***minnesotensis***.

A, a spore of AS3G1 attached to *C*. *elegans* second stage larvae (L2); B, formation of an infection peg; C, the apex of the infection peg becoming globose; D, initial colonization of coelom by hyphae; E–G, hyphal growth through coelom; H–J, hyphal growth out of coelom; K–L, formation of new conidia. The scale bar in A–F, H–I, and L corresponds to 20 μm, whereas the scale bar in G, J and K equals 40 μm.

should read:

**Progression of** ***H. glycines*** **J2 infection by** ***H***. ***minnesotensis***

A, a spore of AS3G1 attached to *H. glycines* second stage juvenile (J2); B, formation of an infection peg; C, the apex of the infection peg becoming globose; D, initial colonization of coelom by hyphae; E–G, hyphal growth through coelom; H–J, hyphal growth out of coelom; K–L, formation of new conidia. The scale bar in A–F, H–I, and L corresponds to 20 μm, whereas the scale bar in G, J and K equals 40 μm.

